# Protection of medical personnel in armed conflicts—case study: Afghanistan

**DOI:** 10.1007/s00068-013-0251-0

**Published:** 2013-02-09

**Authors:** M. Goniewicz, K. Goniewicz

**Affiliations:** 1 Faculty of Health Sciences, School of Economics and Law in Kielce, Jagiellońska 109a, 25-734 Kielce, Poland; 2 University of Economics and Innovation, ul. Mełgiewska 7-9, 20-209 Lublin, Poland

**Keywords:** Protection of medical personnel, Law of armed conflict, Military medicine

## Abstract

**Introduction:**

International humanitarian law affords special protection to medical property and personnel whose mission is to save lives and provide health care for civilians and combatants alike.

**Discussion:**

This paper presents the legal aspects of medical-personnel protection in armed conflicts. Presented below are examples of the Afghanistan analyses where, as a result of war situations, people are most vulnerable. Discussed are the minimum protection and standards applicable to such situations specified by the international humanitarian law.

**Conclusion:**

Its rules and provisions obligate fighting parties to take all necessary measures to protect and respect medical missions in all circumstances.

## Introduction

As contemporary armed conflicts become more common, the lack of respect for the signs of the Red Cross and Red Crescent, under which medical services operate, is noticeable. As a result of the treachery and abuse of pictographic representations of the Red Cross and Red Crescent, it has reached a point where the people and objects entitled to be protected have become as much of a target as military units. Despite the undeniable recognition of these protective symbols, the parties involved in the conflict cannot always be certain whether the signs of the Red Cross or the Red Crescent are being used by people with authorization. This situation is widespread in the conflict in Afghanistan, where the usage of these symbols is inconsistent with international conventions, and has led to numerous events where medical vehicles marked with the Red Cross or the Red Crescent symbols, together with their employees, are often targets of terrorist attacks. Additionally, similar reactions have been encountered by the units of the International Committee of the Red Cross (ICRC) when providing humanitarian help to citizens of Afghanistan. This is considered a violation of the Geneva Conventions, which prohibit attacks on people and objects bearing the emblem of protection. However, it automatically raises the problem of obtaining and/or guaranteeing that the marked unit* is* actually an ambulance or a humanitarian mission, not a branch of the armed forces of the enemy. This situation applies to both verifying the credibility of the mark and its protection, and protecting the rights established in order to save human life and health. All of these issues influence the work of the ICRC, but mainly they affect the neediest, who are involved in situations of armed conflict and are waiting for help from humanitarian organizations and emergency medical services.

## Protection of medical services and victims of armed conflict under the Geneva Conventions

The international humanitarian law of armed conflict emerged in the nineteenth century. The first piece of legislation regulating this issue was signed on the 22nd of August 1864: the Convention for the Amelioration of the Condition of the Wounded and Sick in Armies in the Field [1]. The Convention not only introduced an important international obligation to comply with humanitarian principles, but it correspondingly created the basis for contemporary humanitarian law. Moreover, it formulated standards aimed at protecting victims of armed conflict—particularly wounded soldiers. The Convention reaffirmed the neutrality of medical services and obliged others to respect their actions. The Convention defined the rules pertaining to the usage of and the reverence that should be shown to the Red Cross emblem on a white background [2].

A valuable feature of the Convention was its multilateral nature, which made it possible for all interested countries to accede to it. In 1906, the Convention was replaced by a new agreement, which was also signed in Geneva. That new Convention extended the scope of protection regarding wounded and injured soldiers by introducing the principle of keeping records of victims of armed conflicts and established a system for exchanging information about those people [3]. Experiences gained in further conflicts caused the undertaking of additional work on the development of international humanitarian law. By 1949, many different kinds of declarations and conventions came into effect that developed issues outlined in the Geneva Convention of 1906. Afterwards, the Hague Conventions on Laws and Customs of War on Land and the Adaptation to Maritime Warfare of Principles of Geneva Convention of 1864 were signed in 1899. In 1907, some new conventions were introduced (Fourth Hague Convention, Ninth Hague Convention). Then, in 1925, the Geneva Protocol was signed, banning the usage of asphyxiating, poisonous gas or similar substances and bacteriological methods in war. Another two Geneva Conventions were introduced in 1929 to amend the Geneva Convention of 1906 and the Geneva Convention on the treatment of war prisoners.

At the moment, international humanitarian law and the activities of the ICRC during armed conflict are based on four Geneva Conventions of 1949, as well as on the Additional Protocols for these conventions. Combined, these constitute a system of international law that protects victims of armed conflicts [1]. One of the basic principles of this system is that medical personnel and military civilians are entitled to special protection from attacks, and another is that the activities of medical personnel cannot be prohibited or violated. Medical personnel, in accordance with Article 24 of the Geneva Convention, should only be “exclusively engaged in the search for, or the collection, transport or treatment of the wounded or sick, or in the prevention of disease, staff exclusively engaged in the administration of medical units and establishments, as well as chaplains attached to the armed forces, and shall be respected and protected in all circumstances.” Geneva Convention II grants protection to medical personnel, as well as to personnel of hospital ships. According to Article 36 of Geneva Convention II, these personnel “shall be respected and protected; they may not be captured during the time they are in the service of the hospital ship, whether or not there are wounded and sick on board.” Article 37 constitutes that if religious, medical, and hospital personnel fall into the hands of the enemy, they must be respected and protected; they may continue to carry out their duties as long as necessary for the care of the wounded and sick. They shall afterwards be sent back as soon as the Commander-in-Chief, under whose authority they are operating, considers it practicable. They may take their personal property with them upon leaving the ship.

The First Additional Protocol to the Geneva Convention defines that civilian medical personnel shall be respected and protected and, if needed, all available help shall be afforded to civilian medical personnel in an area where civilian medical services are disrupted due to combat activity. The occupying power shall afford civilian medical personnel in occupied territories any assistance required in order to enable them to perform, to the best of their ability, their humanitarian functions. When they are performing those functions, the occupying power cannot compel these personnel to give priority to the treatment of any person, except on medical grounds. The personnel cannot be compelled to carry out tasks that are not compatible with their humanitarian mission. Civilian medical personnel shall have access to any place where their services are essential, subject to such supervisory and safety measures as the relevant party to the conflict may deem necessary. Civilian religious personnel shall be respected and protected. The provisions of the Conventions and of this Protocol concerning the protection and identification of medical personnel shall apply equally to such persons.

According to Article 16 regarding the general protection of medical duties, under no circumstances shall any person be punished for carrying out medical activities compatible with medical ethics, regardless of the person benefiting from them. Persons engaged in medical activities shall not be compelled to perform acts or to carry out work contrary to the rules of medical ethics or to other medical rules designed for the benefit of the wounded and sick, or to the provisions of the Conventions or of this Protocol, or to refrain from performing acts or from carrying out work required by those rules and provisions. No person engaged in medical activities shall be compelled to give to anyone belonging either to an adverse party, or to his own party (except as required by the law of the latter party) any information concerning the wounded and sick who are (or who have been) under his care, if such information would—in his opinion—prove harmful to the patients concerned or to their families. Regulations for the compulsory notification of communicable diseases shall, however, be respected. Article 38 of Protocol I, additional to the Geneva Conventions, prohibits the unauthorized use of the Red Cross image and other emblems, signs, and signals recognized internationally. Article 9 of Protocol II, also additional to the Geneva Conventions, explores the issue of protection regarding medical personnel. Furthermore, according to this article, all possible help must be provided to enable medical personnel to fulfill their functions. Medical personnel should also not be forced to perform tasks that are impossible to reconcile with their humanitarian mission. Moreover, they should not be asked to give priority to any person for reasons other for than medical purposes. Article 10 of Protocol I, additional to the Geneva Conventions, establishes general rules concerning the protection of medical duties. Due to the general protection of medical duties, under no circumstances shall any person be punished for having carried out medical activities that are compatible with medical ethics, regardless of the person benefiting from them. Persons engaged in medical activities shall neither be compelled to perform acts or to carry out work contrary to nor be compelled to refrain from acts required by the rules of medical ethics or other rules designed for the benefit of the wounded and sick, or this Protocol. The professional obligations of persons engaged in medical activities regarding information which they may acquire concerning the wounded and sick under their care shall, subject to national law, be respected. Subject to national law, no person engaged in medical activities may be penalized in any way for refusing or failing to give information concerning the wounded and sick who are, or who have been, in their care. Article 11 treats the issue of protection of medical units and transports, which shall be respected and protected at all times and shall not be the objects of attack. The protection to which medical units and transports are entitled shall not cease unless they are used to commit hostile acts outside of their humanitarian function. Protection may, however, cease only after a warning has been given, setting—whenever appropriate—a reasonable time limit, and after such a warning has remained unheeded.

Nowadays, the Geneva Conventions apply in all cases where hostilities are ongoing, regardless of whether the war is declared or not. Moreover, the classification of the armed conflict by its participants does not matter. In addition to war, the Geneva Conventions are concerned with occupation, even if there is no armed resistance. Furthermore, the Conventions apply to all states, including situations where one of the countries involved in the conflict may not be a party to the Convention [4]. In accordance with Articles 47, 48, 127, and 144 of Geneva Conventions I, II, III, and IV, respectively, it is a legal obligation of countries to spread knowledge of these Conventions and Protocols [5].

Since 1949, the Geneva Conventions have been extended by a number of other regulations, conventions, agreements, and protocols, which have significantly influenced the current shape of international humanitarian law. Contemporary humanitarian law is defined as the norms of conduct devoted to humanity, human dignity, life, and health; the norms adopted jointly by the countries and adopted widely in international law; legal standards that take the form of conventions and other international agreements, as well as those included by custom; norms of international law applicable throughout the world in times of peace, war, and other circumstances; and a set of rules of international law, enacted and adopted in order to provide assistance and care for human beings, in particular the Geneva Conventions of 12 August 1949 for the protection of war victims and the Additional Protocols of 1977 to those Conventions.

## Protection of medical services and victims of armed conflict according to the standards of the International Red Cross and Red Crescent Societies and Human Rights

Humanitarian law is the basis for the International Red Cross and Red Crescent Societies. Moreover, the Red Cross is a promoter of humanitarian law. In order to facilitate the dissemination of knowledge regarding international humanitarian law, the ICRC has developed a set of standards that constitutes the essence of international humanitarian law. They have no legal force and do not replace the applicable international treaties. These norms, which are customary in nature, state that nonparticipants or people that are not able to partake in a fight are entitled to respect for their lives and their physical and mental integrity. Such individuals must always be protected and treated humanely, without any discussion; it is forbidden to kill or inflict wounds on an enemy who surrenders or can no longer take part in a fight; wounded and sick must be gathered and taken into the care of the party in the conflict who they are collected by. Additionally, medical staff and medical devices, vehicles, and equipment are also subject to this protection; apprehended combatants and civilians that are under the power of the opposing party are entitled to respect for their lives, dignity, personal rights, and their political, religious, and other opinions; everyone must adhere to the basic judicial guarantees, and no one can be held responsible for an act they did not commit. No one shall be subjected to physical or mental torture or corporal punishment or any other cruel or degrading treatment; parties to the conflict and members of their armed forces do not have the unlimited right to choose methods and means of warfare. It is forbidden to use weapons or methods of warfare that may cause unnecessary losses or excessive suffering; parties to the conflict shall at all times distinguish between the civilian population and combatants in order to spare the civilian population and their property. The civilian population as a whole and individual civilians shall not be the objects of attack. Attacks may only be directed against military objects [6].

Humanitarian law is implemented in a situation of armed conflict. It is designed to provide assistance and protection to all people and to reduce the suffering caused by war. Moreover, humanitarian law provisions regulate relations with the enemy, the management of war prisoners, and the rights of residents of a territory occupied by a foreign country. However, humanitarian law does not address the legality and illegality of armed conflict. The preamble to the Protocol I (additional to the Geneva Conventions) contains the following statement: “(…) Reaffirming further that the provisions of the Geneva Conventions of 12 August 1949 and of this Protocol must be fully applied in all circumstances to all persons who are protected by those instruments, without any adverse distinction based on the nature or origin of the armed conflict or on the causes espoused by or attributed to the Parties to the conflict (…)” [7].

Human rights are in no way concerned with the methods used in military operations (for example, the methods of use of weapons). Furthermore, they apply in times of both peace and war. Their purpose is to protect individuals; to facilitate the development and strengthening of the human being in opposition to a government. Only in exceptional circumstances and in specific cases (as described in acts of international and national law) is it possible to ignore some of its provisions [8]. In international regulations dealing with human rights issues, there have been provisions that authorize the state to suspend these rights in a situation threatening its existence [9]. Nevertheless, certain fundamental rights mentioned in all international treaties are treated as exceptions. They are considered the “core rights” that cannot be suspended under any circumstances. This applies in particular to the right to live, the prohibition of torture and inhuman behavior, the prohibition of slavery and servitude, as well as the principle of the legality and nonretroactivity of the law. Most of the rights included in Article 4 of the International Covenant on Civil and Political Rights may be repealed in the case of armed conflict.

Conversely, the following rights can never be repealed: prohibition of death penalty rulings except in court proceedings and some limitations to this penalty; prohibition of torture and inhuman or degrading treatment; prohibition of slavery and servitude; prohibition of retroactivity of new or stricter rules of substantive criminal law; the right to own a legal personality forever; the rights to freedom of thought, conscience, and religion [9].

According to the ICRC: “(…) The mechanisms controlling obeying human rights are very diverse. In many cases, relevant institutions are responsible to determine whether a breach of the law has taken place or not. For example, the European Court of Human Rights may, after completion of the procedure in a particular case, state that the authorities of the country violated the European Convention on Human Rights. Afterwards, the authorities have the obligation to implement the necessary measures to ensure that the internal situation comply with the standards set out in the Convention. All in all, mechanisms responsible for the implementation of human rights are essentially designed to allow compensation for the harm suffered” [10]. Additionally, human rights applicable in the context of armed conflict are complemented by international humanitarian law. For that reason, both systems aim to ensure the protection of the human being, but through the use of other means and in other circumstances [11].

## Protection of medical services and victims of armed conflict in the light of international and non-international armed conflict

The war in Afghanistan was recognized as an international armed conflict in the beginning. Consequently, in the course of the conflict, the Geneva Conventions and Additional Protocols have applied. Humanitarian law has been applied to the parties of the conflict, but has also provided protection to people and groups who have not participated in the conflict or who have ceased to take part in it. In accordance with the Additional Protocol provisions by special care, the following are also covered: wounded and ill soldiers in terrestrial conflicts, as well as members of the medical services of the armed forces; wounded, ill, or shipwrecked soldiers in the war at sea, as well as members of the naval medical service; prisoners of war; and the civilian population, such as foreign civilians who are present on territory belonging to the parties in the conflict, including refugees, civilians in occupied areas, arrested and interned civilians, medical and religious personnel, and civil defense units [4].

Many of the standards contained in the Additional Protocols to the Geneva Conventions referring to international conflicts are treated as a norm of customary law applicable in all armed conflicts. This is important because Additional Protocol I discusses the issue of protecting civilians and the measures necessary to shield them from the effects of hostilities.

According to the Nuremberg Tribunal: “The law of war is not only contained in treaties, but also in the habits and customs, which gradually have gained general recognition, as well as the general principles of justice applied by jurists and military courts” [12]. As a result, the law is not static, but is adapted by continual embellishments to the needs of a changing world. However, in many cases the treaty merely expresses and defines existing legal principles in more detail.

In the international humanitarian law of armed conflict, material aid to the victims of conflict is ensured. According to these standards, each high contracting party shall allow the free passage of all consignments of medical and hospital stores and objects necessary for religious worship intended only for civilians of another high contracting party, even if the latter is its adversary. It shall likewise permit the free passage of all consignments of essential foodstuffs, clothing, and tonics intended for children under fifteen, expectant mothers, and maternity cases. To the fullest extent of the means available to it, the occupying power has the duty to ensure the food and medical supplies of the population; it should, in particular, bring in the necessary foodstuffs, medical stores, and other articles if the resources of the occupied territory are inadequate. If the whole or part of the population of an occupied territory is inadequately supplied, the occupying power shall agree to relief schemes on behalf of the said population, and shall facilitate them by all of the means at its disposal. In all cases, the duration of the period during which a protected person accused of an offence is under arrest awaiting trial or punishment shall be deducted from any period of imprisonment awarded.

Protected persons shall not be arrested, prosecuted, or convicted by the occupying power for acts committed or for opinions expressed before the occupation, or during a temporary interruption thereof, with the exception of breaches of the laws and customs of war. Nationals of the occupying power who sought refuge in the territory of the occupied state before the outbreak of hostilities shall not be arrested, prosecuted, convicted, or deported from the occupied territory, except for offences committed after the outbreak of hostilities, or for offences under common law committed before the outbreak of hostilities which, according to the law of the occupied state, would have justified extradition in peacetime.

At this stage, the military encounter in Afghanistan should be classified as a non-international armed conflict due to the fact that it takes place between Afghan guerrillas, which are not the government’s armed forces and NATO troops. Moreover, it is noteworthy that the conditions for the implementation of Additional Protocol II are stricter than the conditions needed for the application of Article 3 of the Geneva Conventions. Furthermore, in such situations, humanitarian law applies to the armed forces—both regular and irregular—that are involved in the conflict, and it protects any person or class of people who do not participate in hostilities or have ceased to take part in them (i.e., wounded and sick combatants, individuals deprived of their liberty as a result of the conflict, civilians, and medical and religious personnel).

On the other hand, during a non-international armed conflict, humanitarian law provides material aid to victims of the struggle. In Article 18 of Additional Protocol II, it is stated that: “If the civilian population is suffering undue hardship owing to a lack of the supplies essential for its survival, such as food-stuffs and medical supplies, relief actions for the civilian population which are of an exclusively humanitarian and impartial nature and which are conducted without any adverse distinction shall be undertaken subject to the consent of the High Contracting Party concerned” [13].

Furthermore, the conflict in Afghanistan is not only a war against the Taliban but a combination of several minor conflicts that involve—in addition to an international coalition gathered under the banner of the ISAF—all sorts of entities (both state and private), international terrorist organizations, and criminals. This would also include different tribes, mercenaries, religious and ideological leaders, and intelligence services that have broken away from the control of the state. Therefore, ending the conflict in Afghanistan is an extremely difficult task, and probably one that will prove impossible to achieve for a long time. Additionally, in the case of a “new war,” it is very important that international humanitarian law is not toothless and is fully applied.

## Conclusions

All in all, in the history of armed conflict there have been many examples of neglect and failure to comply with international humanitarian law, relating to members of the armed forces, medical personnel, humanitarian workers (ICRC), as well as civilians. This is because of a lack of respect for the signs of the Red Cross and Red Crescent under which the medical professionals operate, the attitudes of governments that ignore the action of international organizations and institutions on the grounds that this action constitutes interference in their internal affairs (such as the armed conflict in Darfur, the western province of Sudan), and the occurrence of a new kinds of armed conflict, including so-called unstructured conflicts (French “destructurés”—there is no clear division between the warring parties) and fights between armed forces and terrorists in circumstances of large-scale terrorist activity (such as the Russian–Chechen conflict). In addition, modern international humanitarian law provides protection to devices (means of transport, hospitals) and medical staff. Consequently, in situations of international armed conflict, the Geneva Conventions and Additional Protocol I should be implemented. In this type of conflict, humanitarian law is intended to primarily protect the parties of the conflict, as well as any individuals or any group of people who do not participate in the conflict or have ceased to take part in it. In the case of a non-international armed conflict, Article 3 common to the four Geneva Conventions and Additional Protocol II is applied.

Nevertheless, in line with contemporary international humanitarian law in armed conflicts (especially non-international conflicts, in which there is no clear division between the warring parties) where the application of military force is legally and morally justified, there are certain measures that cannot be executed. The validity of the fight against terrorism, “the scourge of the twenty-first century” does not justify the use of certain forms of violence, especially against civilians. For that reason, attacking civilians, including medical personnel, is a violation of the Geneva Conventions; it is a war crime and a crime against humanity.

Thus, all countries that have ratified the Geneva Conventions and Additional Protocols are obliged to follow the rules of war outlined in them and to ensure their dissemination during peacetime. Educating societies in the field of international humanitarian law may help to prevent attacks on medical facilities and personnel, as well as significantly improve the fate of the victims of armed conflict.
